# Late presentation of posterior urethral valve: two case reports

**DOI:** 10.1590/S1516-31802008000200012

**Published:** 2008-03-06

**Authors:** Carlos Márcio Nóbrega de Jesus, José Carlos de Souza Trindade, José Goldberg

**Keywords:** Abnormalities, Urethra, Adult, Adolescent, Diagnosis, Anormalidades, Uretra, Adulto, Adolescente, Diagnóstico

## Abstract

**CONTEXT:**

Posterior urethral valve (PUV) is a widely known condition affecting males that generally presents prenatally or at birth. PUVs have also been occasionally described in literature in cases diagnosed during adolescence or adulthood.

**CASE REPORT:**

This report presents two late PUV cases, one in a teenager and the other in an adult. Both cases had had clinical signs of urinary tract infection and obstructive urinary symptoms. The diagnoses were made by means of voiding cystourethrography and urethrocystoscopy. Endoscopic valve fulguration was the treatment chosen for both. Their follow-up was uneventful.

## RESUMO

**CONTEXTO:**

Válvula de uretra posterior (VUP) é uma conhecida malformação congênita urinária, geralmente diagnosticada em exames ultra-sonográficos pré-natais ou ao nascimento. Raramente, esta doença pode ser encontrada em adolescentes e em adultos.

**RELATO DE CASOS:**

Este artigo mostra dois casos de VUP, encontrados em um adolescente e em um adulto. Ambos apresentavam sinais clínicos de infecção do trato urinário e sintomas obstrutivos infravesicais. Os diagnósticos foram realizados por uretrocistografia miccional e uretrocistoscopia. Fulguração endoscópica das válvulas foi o tratamento de escolha para ambos os casos. O acompanhamento demonstrou melhora importante dos sintomas após o tratamento.

## INTRODUCTION

Posterior urethral valves (PUVs) are the most common cause of lower urinary tract obstruction in male neonates, with an incidence of one case per 8,000 to 25,000 live births.^1,2^ The diagnosis is usually made prenatally or at birth, when male newborns are evaluated for prenatal hydronephrosis, or during early childhood, but rarely during adolescence or adulthood. The clinical features at these later stages can be confused with many other diseases, thus making correct diagnosis difficult. Here, we describe two cases of late presentation of PUV, which were suspected due to urinary tract infection and voiding dysfunction.

## CASE REPORT

### Case 1

**Figure 1.1:**
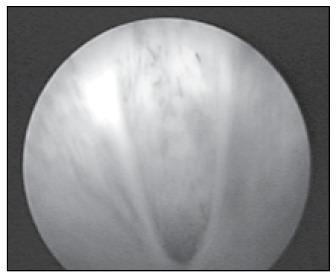
Voiding cystourethrography showing dilation and wide urethra in an 11-year-old boy.

**Figure 1.2:**
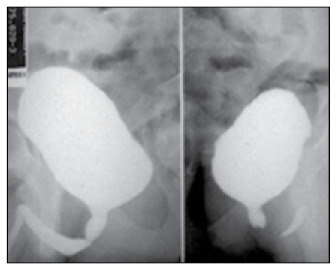
Urethrocystoscopy showing type I urethral valves in an 11-year-old boy.

An 11-year-old white boy presented intermittent low-pressure voiding with dysuria for one month. He had presented nocturnal enuresis until he was eight years old. His urine sample showed bacteriuria and large numbers of degenerated leukocytes and was positive for nitrite. Urine culturing was positive for *Escherichia coli*. After appropriate treatment with antibiotic therapy, this clinical condition appeared again, one month later. Renal and bladder ultrasound scans were normal. During voiding cystourethrography (VCU), dilation and a wide prostatic urethra with a "stop" in the membranous urethra were observed (Figure 1.1). There was no vesicoureteral reflux. The correct diagnosis was made by cystoscopy, which confirmed the presence of type I urethral valves (Figure 1.2). Endoscopic valve fulguration was the treatment chosen. This patient evolved successfully and he has not presented any recurrence of urinary symptoms so far.

### Case 2

**Figure 2:**
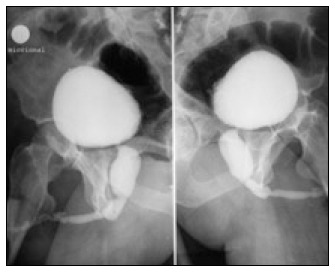
Voiding cystourethrography showing dilation and wide urethra, and several urethral stenoses, in a 40-year-old adult who had had a history of weak urine stream since childhood.

A 40-year-old white man had a history of recurrent urinary tract infection, and his urethral discharge had been accompanied by poor, dribbling urine stream since childhood. He sought medical assistance due to a worsening of his urinary symptoms. Digital prostate examination gave normal results. Urine culturing was positive for Escherichia coli. Serum creatinine was within normal limits. Renal ultrasound did not show hydronephrosis. In addition to many stenoses, VCU showed a dilated large posterior urethra and a thick and irregular bladder wall (Figure 2). During endoscopic urethrotomy, type III diaphragmatic posterior urethral valves were observed and fulgurated. This patient is now in a good condition with satisfactory urinary stream.

### DISCUSSION

Late presentation of PUV is a rare condition and it has been estimated that it accounts for 10% of PUV cases.^3^ The usual presentation is prenatal or at birth. Poor or weak stream, dribbling voiding, repeated urinary tract infection and chronic renal failure are the most common clinical pictures in adolescents and adults.^4^ In most cases, PUV fulguration improves the signs and symptoms of obstructive infravesical syndrome while preserving renal function and renal morphology.^5^

Many common conditions are superimposed on late PUV, such as benign prostate hypertrophy, urethral stenosis, prostatitis, urethritis and sphincter-bladder dyssynergy. These other pathological conditions delay or confound the diagnosing of late PUV and, for this reason, we had to consider this condition when the second patient told us about his weak stream and history of urinary tract infection since childhood. Within this scenario, it is essential to include VCU among the diagnostic tools.

Other rare situations could be related to late presentation of PUV, such as retained ejaculation,^6^ infertility,^7^ enuresis^8^ or perineal pain with dilated Cowper’s glands.^9^ However, late presentation of PUV could be a cause of chronic renal failure whether or not it has been treated. Parkhouse at al.^10^ reported on long-term follow-up among treated postpubertal patients and showed that 26% of them had had chronic or end-stage renal failure.^10^ Today, 78 late PUV cases have been described in the literature. We believe that this number is smaller than might have been imagined, because of diagnostic difficulties and the existence of other, similar diseases. In other words, urologists must remain alert to the possibility of late diagnoses of PUV, especially among adults.

In the cases we have reported, the treatment was efficient and led to remission of the illness. However, late PUV is a condition that puts such patients at risk. In a retrospective review of 47 patients who presented late PUV, Bomalaski et al. reported the presence of elevated serum creatinine in 35% and end-stage renal disease in 10%.^4^ This shows that such patients are at risk of progression to end-stage renal disease. Therefore, these patients must be treated despite the mild signs and symptoms exhibited when PUV is diagnosed as a late presentation.

## CONCLUSION

In conclusion, PUV is not merely a disease of infancy. Boys and men must be investigated if they present voiding complaints, in order to rule out this pathological condition. Urologists must bear this diagnosis in mind and remember that its presentation may be more frequent than might be imagined.
